# Lotus Root-Like Nitrogen-Doped Carbon Nanofiber Structure Assembled with VN Catalysts as a Multifunctional Host for Superior Lithium–Sulfur Batteries

**DOI:** 10.3390/nano9121724

**Published:** 2019-12-03

**Authors:** Benben Wei, Chaoqun Shang, Xiaoying Pan, Zhihong Chen, Lingling Shui, Xin Wang, Guofu Zhou

**Affiliations:** 1 International Academy of Optoelectronics at Zhaoqing, South China Normal University, Zhaoqing 526238, China; benben.wei@ecs-scnu.org (B.W.); guofu.zhou@m.scnu.edu.cn (G.Z.); 2 National Center for International Research on Green Optoelectronics, South China Normal University, Guangzhou 510006, China; xiaoying.pan@ecs-scnu.org (X.P.); shuill@m.scnu.edu.cn (L.S.); 3 Key Laboratory for Water Quality and Conservation of the Pearl River Delta, Guangzhou University, Guangzhou 510006, China; chenzhihong1227@sina.com

**Keywords:** lithium–sulfur batteries, polysulfide shuttling, nanostructure, catalytic, VN

## Abstract

Lithium–sulfur batteries (LSBs) are regarded as one of the most promising energy-recycling storage systems due to their high energy density (up to 2600 Wh kg^−1^), high theoretical specific capacity (as much as 1672 mAh g^−1^), environmental friendliness, and low cost. Originating from the complicated redox of lithium polysulfide intermediates, Li–S batteries suffer from several problems, restricting their application and commercialization. Such problems include the shuttle effect of polysulfides (Li_2_S_x_ (2 < x ≤ 8)), low electronic conductivity of S/Li_2_S/Li_2_S_2_, and large volumetric expansion of S upon lithiation. In this study, a lotus root-like nitrogen-doped carbon nanofiber (NCNF) structure, assembled with vanadium nitride (VN) catalysts, was fabricated as a 3D freestanding current collector for high performance LSBs. The lotus root-like NCNF structure, which had a multichannel porous nanostructure, was able to provide excellent (ionically/electronically) conductive networks, which promoted ion transport and physical confinement of lithium polysulfides. Further, the structure provided good electrolyte penetration, thereby enhancing the interface contact with active S. VN, with its narrow resolved band gap, showed high electrical conductivity, high catalytic effect and polar chemical adsorption of lithium polysulfides, which is ideal for accelerating the reversible redox kinetics of intermediate polysulfides to improve the utilization of S. Tests showed that the VN-decorated multichannel porous carbon nanofiber structure retained a high specific capacity of 1325 mAh g^−1^ after 100 cycles at 0.1 C, with a low capacity decay of 0.05% per cycle, and demonstrated excellent rate capability.

## 1. Introduction

Lithium–sulfur batteries (LSBs) have attracted tremendous interest in the energy storage field. LSBs are considered a promising potential energy storage system for the future because of their evident advantages, including high energy and power density, environmental friendliness and low cost as a result of the sulfur-rich nature of Earth [1,2,3]. However, commercialization of LSBs has been impeded by low S utilization, a consequence of the shuttle effect of soluble lithium polysulfides, along with fast capacity decay and low electron conductivity of S/Li_2_S_2_/Li_2_S, huge volume variation of active S upon lithiation, and corrosion of Li anodes [4,5,6,7]. In recent years, various strategies have been adopted to weaken the influence of the above issues and improve the electrochemical performance of LSBs, including modification of the separator [8,9,10,11,12,13,14], the creation of new electrolytes [15,16,17,18], the addition of protection for the Li anode [19,20,21], and improvement of the sulfur host [22,23,24,25,26,27,28]. Among these, the creation of a novel sulfur host is a promising tactic to promote sulfur utilization and buffer volume expansion in the cathode.

Nanostructured porous carbon materials were first investigated as lithium polysulfide traps by way of physical confinement. Such materials as graphene, carbon nanosheets, carbon spheres, carbon nanotubes, and microporous carbon materials, provide excellent electron transport networks for sulfur intermediates of the redox reaction and high specific surface areas to accommodate volumetric expansion [29,30,31,32,33,34,35,36,37,38,39]. Enhancement of lithium polysulfide redox kinetics requires electrochemically available polysulfides once the polysulfides are adsorbed on the carbon materials. However, soluble and polar lithium polysulfides can lose their electrical contact with nonpolar carbon materials and dissolve into the electrolyte after a long work time as a consequence of weak polar–nonpolar effects, leading to poor Coulombic efficiency, low utilization of S and slow redox kinetics [40,41]. Thus, polar host materials, such as transition metal oxides and sulfides, are employed to improve the adsorption of lithium polysulfides by enhancing the chemical binding [42,43,44,45,46,47,48,49]. Unfortunately, unsatisfied electrical conductivity of most metal oxides and sulfides is detrimental to the kinetics of sulfur electrochemical conversion, leading to poor cycling performance and low sulfur utilization, as well as poor rate capability [50,51,52,53]. In comparison, transition metal nitrides possess good electrical conductivity for promotion of lithium polysulfide conversion, which can alleviate the dissolution of the cathode into the electrolyte and facilitate cycling stability [54,55,56,57,58,59,60,61,62]. As one of the transition metal nitrides, vanadium nitride (VN), with its high electrical conductivity (1.17 × 10^6^ S m^−1^ at room temperature), can be a good choice to effectively anchor lithium polysulfide intermediates and to restrict the shuttle effect as a polar host, thereby improving sulfur utilization and enhancing cycling stability through high electron mobility and the catalytic effect [63,64,65,66,67].

In this study, we prepared a lotus root-like nitrogen-doped carbon nanofiber (NCNF) structure, assembled with VN catalysts, to act as a self-supported current collector in LSBs. With more reaction sites, the freestanding 3D nanostructure enhanced the contact area with the electrolyte, providing good electron and ion transport networks. The VN/NCNF structure was found to be capable of trapping lithium polysulfides by both physical confinement and chemical affinity, and accelerate conversion of lithium polysulfides to enhance the energy storage of LSBs. Finally, a highly reversible capacity of 1325 mAh g^−1^ at 0.1 C with a low capacity decay of 0.05% per cycle was obtained, as well as a favorable rate capability. 

## 2. Experiment

### 2.1. Preparation of VN Current Collector

VN-decorated CNFs were fabricated by electrospinning. Under magnetic stirring (70 °C), 1.2 g of polyacrylonitrile (PAN), 0.3 g of polystyrene (PS), 0.3 g of polyvinyl pyrrolidone (PVP) and 0.9 g of vanadium (IV) oxy(acetylacetonate) (VO(acac)_2_) were mixed with 15 mL DMF overnight, forming the electrospun solution. At an environmental temperature of 50 °C, electrospinning was carried out with a 19G needle, distance of 20 cm to the Al-foil collector, 2.4 mL h^−1^ feed rate and 13.5 kV applied voltage. The collected nanofiber disks (diameter of 19 mm) were annealed at 280 °C (2 h, 5 °C min^−1^) and subsequently 800 °C (5 h, 2 °C min^−1^) under Ar. After annealing, VN-decorated multichannel porous CNF (denoted as MPVN) disks with diameters of approximately 9 mm were obtained. MVN (VN-decorated multichannel CNF), PVN (VN-decorated porous CNF) and NCNF (nitrogen doped CNF) were also obtained with same procedure without PVP, PS or VO(acac)_2_, respectively. 

### 2.2. Li_2_S_6_ Adsorption and X-ray Photoelectron Spectroscopy (XPS) Sample Preparation

Li_2_S_6_ catholyte was prepared by dissolving sublimed sulfur and lithium sulfide with a molar ratio of 5:1 in an electrolyte solution (1.0 M LiTFSI in a 1:1 (v/v) 1,3-dioxolane (DOL)/dimethyl ether (DME) mixture with 1.0 wt % LiNO_3_) by stirring (60 °C, 48 h). To study the adsorption capability of VN, the same weight (20 mg) of MPVN, MVN and PVN were mixed with 10 uL of 1 M Li_2_S_6_ catholyte and 1mL electrolyte (as described above). After 8 h of adsorption, the solution was removed, and MPVN, MVN and PVN with adsorbed Li_2_S_6_ were dried for XPS analysis. All of the above operations were carried in a glovebox filled with argon (with O_2_ < 0.01 ppm, H_2_O < 0.01 ppm).

### 2.3. Assembly of Li–S Batteries and Symmetric Batteries

Coin cells (2032-type) were assembled using lithium metal (counter electrode and reference electrode), a celgard 2500 (separator) and 1.92 mg S (the active material), derived from the 10 μL Li_2_S_6_ (1 M) catholyte. The ratio of electrolyte to S was 15 uL mg^−1^ and the corresponding areal density was 3 mg cm^−2^ (based on sulfur). Symmetric batteries were constructed with VN as the work and counter electrodes and 20 μL of 1 M Li_2_S_6_ catholyte as the active material, wherein the dosage of electrolyte was 10 uL_electrolyte_ mg_s_^−1^. All of the above procedures were performed in a glovebox filled with argon (with O_2_ < 0.01 ppm, H_2_O < 0.01 ppm).

### 2.4. Characterization of Materials and Electrochemical Measurements 

The morphology of the current collector was characterized by a field-emission scanning electron microscope (FESEM; ZEISS Gemini 500, Carl Zeiss Inc., Germany). Images from a transmission electron microscope (TEM), a high-resolution TEM (HR-TEM) and energy-dispersive spectrometer (EDS) mapping were recorded with JEM-2100HR (JEOL Ltd., Tokyo, Japan). X-ray diffraction (XRD) patterns were obtained by BRUKER D8 ADVANCE (Cu K_α_, λ = 0.154056 nm; Bruker, Karlsruhe, Germany) and X-ray photoelectron spectroscopy (XPS) spectra were measured using a Thermos SCIENTIFIC ESCALAB 250Xi (Waltham, MA, USA) with a monochromatic Al K_α_ X-ray source (1486.6 eV; anode operating at 15 kV and 20 mA). Specific surface area (SSA) was derived from the nitrogen adsorption–desorption isotherms (based on Brunauer–Emmett–Teller method) using ASAPA 2020 (Micromeritics Instrument corp., Atlanta, GA, USA). 

Galvanostatic charge–discharge (GCD) electrochemical performance was tested in a NEWARE battery testing system at a confirmed voltage interval, 1.8 to 2.8 V vs Li/ Li^+^, with a current density of 0.5 mA cm^−2^ (0.1 C). Cyclic voltammetry (CV) measurements were performed with a CHI660E electrochemical workstation using a different scan rate for the Li–S batteries (0.05 mV s^−1^) and symmetric batteries (10 mV s^−1^). Electrochemical impedance spectroscopy (EIS) tests were also performed using CHI660E (Chenhua Inc, Shanghai, China) at a potential of about 2.3 V before and after CV, in a frequency range from 100 kHz to 0.01 Hz and an amplitude of 5 mV.

## 3. Results and Discussion

**Figure 1:**
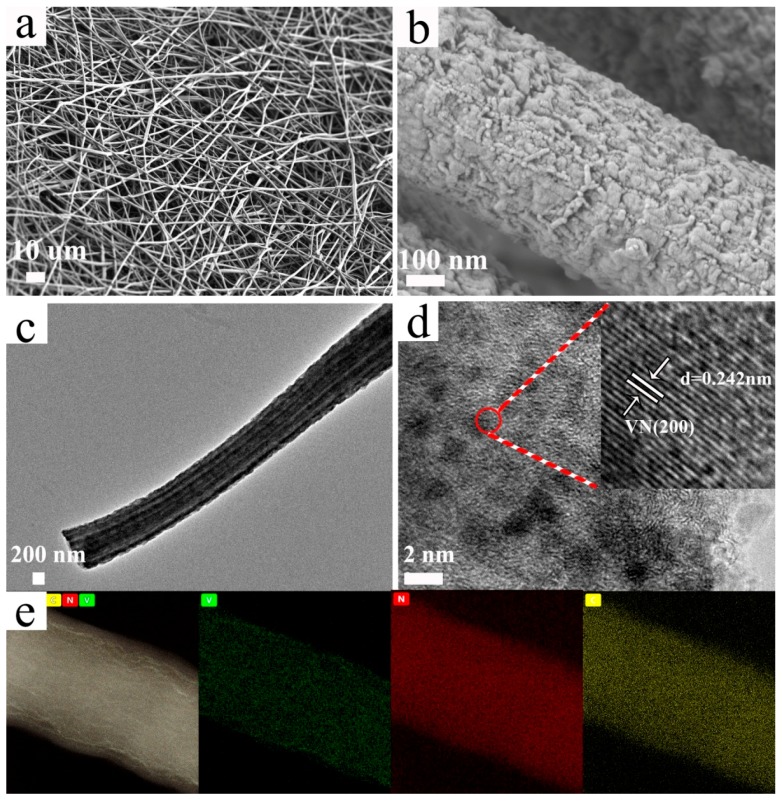
Morphology of VN-decorated multichannel porous carbon nanofibers (MPVN). (**a**,**b**) field-emission scanning electron microscope (FESEM) images, (**c**,**d**) transition electron microscope (TEM) images and (**e**) corresponding energy-dispersive spectrometer (EDS) mapping.

In the electrospun solution, PAN served as the nitrogen source and a continuous template for the droplet phase of PS and PVP because of surface tension differences. During electrospinning, PAN formed a surrounding phase to eliminate Rayleigh instability, entraining discontinuous PS to be a continuous fluid [68]. After stabilization at 280 °C and carbonization at 800 °C, VO(CH_3_COO)_2_ was the in situ nitride, en route to be VN [65,66,67]. Meanwhile, PS and PVP were decomposed to form the multichannel and porous nanostructure, and thus the diameter of the disks decreased from 19 (Figure S1a) to 9 mm (Figure S1b). As shown in Figure 1a, the 3D nanofibers of MPVN created extended interwoven networks, which promote electrolyte penetration and accommodate huge volumetric change during electrochemical reactions. In Figure 1b, the rough surface of a nanofiber can be observed, which may provide enough mesoporous structure to generate reaction sites for lithium polysulfide conversion. TEM images of the nanofibers depicted evident multichannel nanostructure, with a uniform channel diameter of 100 nm, which facilitates ion transport (Figure 1c). In Figure 1d, the HR-TEM image clearly depicts nanosized VN decorating the CNFs, with the inset showing the (200) plane of VN reflected by an obvious lattice space of 0.242 nm, which matches well with VN crystal (face-centered cubic crystal structure with space group of Fm-3m (225)). Homogenous distribution of V, N and C elements was obvious in EDS mapping (Figure 1e), indicating that PAN as a nitrogen source ensured the nitridation of V species and nitrogen doping. The polar nature of VN and nitrogen doping both act to ensure the efficient chemical confinement of lithium polysulfides, while tight contact between the VN-decorated CNFs ensures the fast transformation of lithium polysulfides due to sufficient active sites. To clarify the multichannel and porous nanostructure, MVN and PVN were also prepared without PS or PVP, respectively. MVN, shown in Figure S2, possessed a rough surface and multichannel nanostructure, while PVN, shown in Figure S3, only had a rough surface without multichannel nanostructure. This confirms that the decomposition of PS caused the formation of the multichannel structure and that PVP increased the porosity.

**Figure 2:**
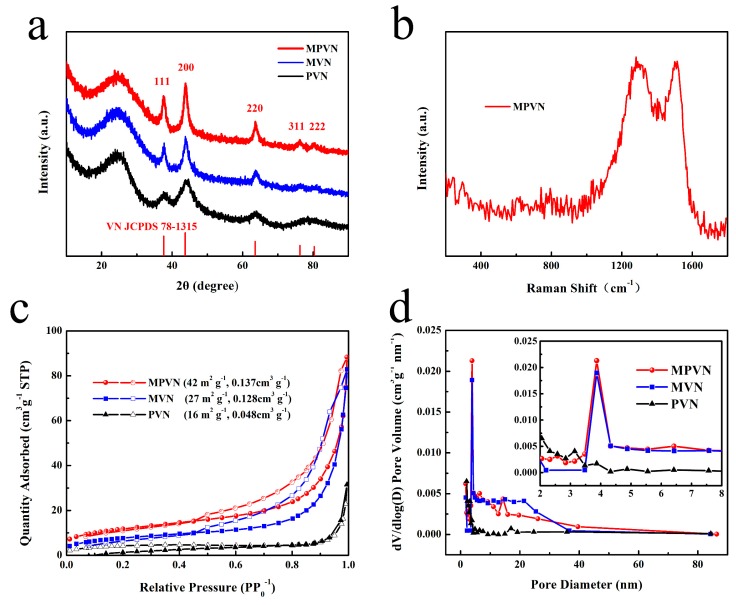
Characterization of materials. (**a**) X-ray diffraction (XRD) results, (**b**) Raman spectrum of MVPN, (**c**) nitrogen adsorption isotherms and (**d**) pore diameter distribution.

The crystal structure of materials was investigated by XRD, as presented in Figure 2a. Well-crystallized VN in MPVN, MVN and PVN was confirmed by diffraction peaks of 37.6°, 43.7°, 63.6°, 76.3° and 80.3°, matching well with the (111), (200), (220), (311) and (222) planes of cubic phase JCPDS 78-1315. Disordered carbon was obvious due to the wide peak found at about 26°. As investigated by Raman spectroscopy, the results of which are presented in Figure 2b, the peaks of MPVN at 1300 and 1500 cm^−1^ may be ascribed to carbon (D band and G band) under 532 nm laser excitation, reflecting disordered and graphitic carbon, respectively. However, the peaks of VN were very weak as a result of the homogenous distribution of VN and the VN peaks being covered by CNF peaks. As depicted in Figure 2c, calculation of nitrogen adsorption–desorption isotherms set SSA at 42 m^2^ g^−1^ and pore volume at 0.137 cm^3^ g^−1^ for MPVN, which was higher than those of MVN (27 m^2^ g^−1^, 0.128 cm^3^ g^−1^) and PVN (16 m^2^ g^−1^, 0.048 cm^3^ g^−1^). As depicted in Figure 2d, MVN had similar mesopore structure to MPVN, further confirming the origin of the multichannel structure from PS decomposition. Compared to MVN and PVN, the increased mesopore structure of MPVN with lotus root-like morphology is beneficial to electron and ion transport, providing sufficient active sites for fast reaction kinetics.

**Figure 3:**
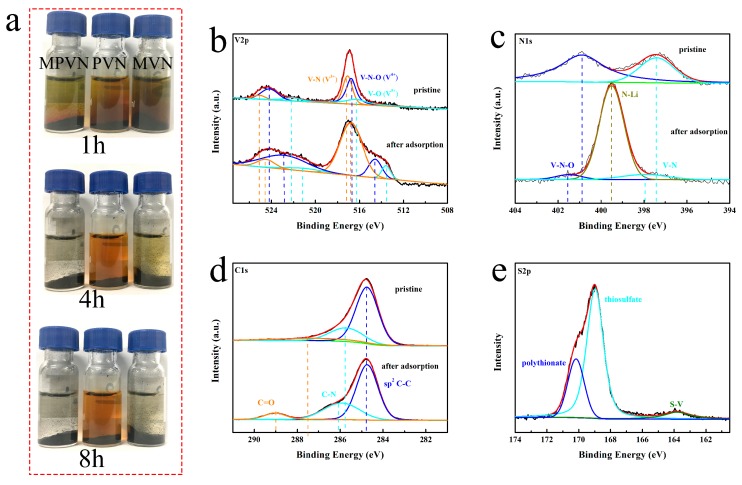
(**a**) Static adsorption of Li_2_S_6_ with MPVN, MVN (VN-decorated multichannel carbon nanofibers) and PVN (VN-decorated porous carbon nanofibers) standing for 1 h, 4 h and 8 h. X-ray photoelectron spectroscopy (XPS) spectra of (**b**) V 2p, (**c**) N 1s, (**d**) C 1s, and (**e**) S 2p in MPVN before and after Li_2_S_6_ adsorption.

The restriction of Li_2_S_6_ dissolution into the electrolyte using MPVN, MVN and PVN was investigated by a visual adsorption test of Li_2_S_6_ solution (Figure 3a). After addition of MPVN, the brown solution was a little lighter after 1h and then completely colorless at 4h. As a fair comparison, MVN made the color of the Li_2_S_6_ solution light after 4 h and colorless after 8 h, while PVN could not completely adsorb the Li_2_S_6_—even after 8 h. The promising adsorption capability of MPVN depends on the synergistic effect of high specific surface area within polar VN. The chemical interaction of VN with lithium polysulfides consists of both Lewis acid–base interaction and polar–polar interaction [69]. As a result of Lewis acid–base interactions, lithium polysulfides can react with VN containing Lewis acid–base react sites to form V–S bonds (Figure 3e), thereby restricting lithium polysulfides from dissolving into the electrolyte [22,69]. As depicted in Figure 3b, three distinguished doublets of V 2p energy bonds were V–N(V^3+^), V–N–O(V^4+^) and V–O(V^5+^) in the MPVN [63]. In addition, the highest intensity peak obtained at 517.10 eV for V–N revealed that the main peak was V^3+^ of VN, further confirming the VN phase in MPVN. After interaction with Li_2_S_6_, the V–N of MPVN underwent more negative shifts (0.34 eV to 516.76 eV) than both MVN (0.21 eV from 517.47 to 517.26 eV), outlined in Figure S4, and PVN (0.19 eV from 517.11 to 516.92 eV), outlined in Figure S5. This indicates a stronger chemical reaction of VN with lithium polysulfides in MPVN, originating from the electron transfer from negative S_x_^2−^ (4 ≤ x ≤ 8) to positive V^3+^ of VN [70]. Benefiting from the polar–polar interaction, long-chain soluble Li_2_S_x_ (4 ≤ x ≤ 8) are anchored on the surface of VN by enhanced polar binding, strengthened during the charging and discharging process. VN depicts excellent catalytic effect to accelerate the conversion of lithium polysulfides and comparable electron conductivity to facilitate electron transport, which induces fast redox kinetics and increases sulfur utilization [71]. Moreover, positive Li ions can interact with negative N anions and N-doping groups with rich electrons to form an N–Li bond (Figure 3c), which further restrains the lithium polysulfide shuttle effect. In the meantime, N–O and N–V of MPVN shift to higher binding energies than those MVN and PVN, indicting the stronger reaction of the N–Li bond in MPVN than MVN and PVN. Figure 3d shows that the C–C bond has almost no binding energy change after reaction with Li_2_S_6_, further demonstrating the poor adsorption of nonpolar carbon to polar lithium polysulfides. In other words, MPVN can be a favorable sulfur host material with efficient restriction of the shuttle effect by strong chemical binding to Li_2_S_x_ (4 ≤ x ≤ 8), thereby ensuring excellent durable cycle stability and high sulfur utilization for superior and stable performance of LSBs. 

**Figure 4:**
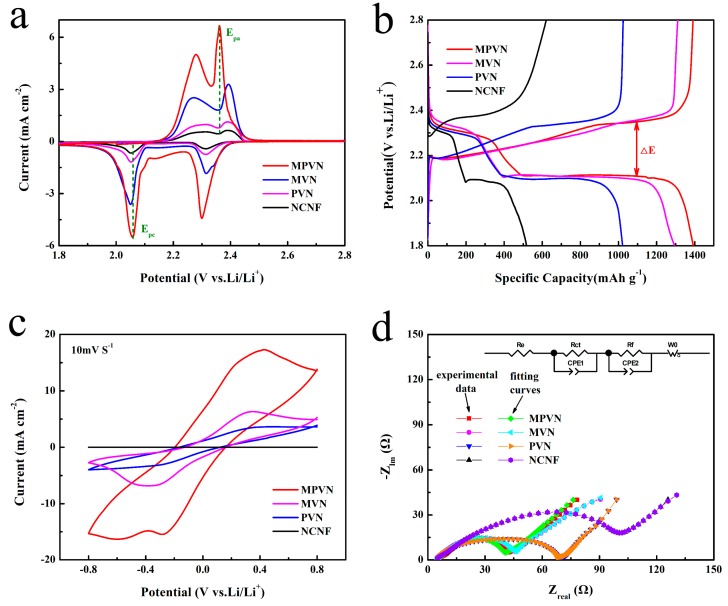
(**a**) Cyclic voltammetry (CV) curves at scan rate of 0.05 mV s^−1^; (**b**) Galvanostatic charge–discharge (GCD) curves of the initial cycles; (**c**) CV curves; and (**d**) Nyquist plots of lithium foil-free symmetric cells.

To demonstrate the catalytic effect of VN towards conversion of lithium polysulfides, the cyclic voltammetry (CV) of MPVN, MVN, PVN and NCNF was measured with sulfur areal loading of 3 mg cm^−2^ at a potential of 1.8 to 2.8 V (vs Li/Li^+^). The CV curves in Figure 4a reveal the typical reduction and oxidation electrochemical reactions of lithium polysulfides through two pairs of distinct peaks. During the charging reaction, the two evident peaks at 2.28 and 2.36 V may be attributed to the oxidation of solid lithium sulfides (Li_2_S_2_/Li_2_S) to soluble long-chain lithium polysulfides (Li_2_S_x_, 4 ≤ x ≤ 8) and to S_8_, respectively. In the cathodic scan, the distinct reduction peaks at 2.31 and 2.06 V belong to a multistep transformation from S_8_ to Li_2_S_2_/Li_2_S. The LSB with MPVN exhibited higher cathodic peak potential (E_pc_) at 2.06 V and lower anodic peak potential (E_pa_) at 2.36 V, with a low polarization of 300 mV, compared to the 340 mV of MVN (E_pc_ at 2.05 V, E_pa_ at 2.39 V), 340mV of PVN (E_pc_ at 2.05 V, E_pa_ at 2.39 V) and 350 mV of NCNF (E_pc_ at 2.05 V, E_pa_ at 2.40 V). Furthermore, the LSB with MPVN exhibited noticeably higher current density. The low polarization and high current density of MPVN indicates excellent VN catalysts, which accelerate the redox kinetics of lithium polysulfides. Impressively, the onset potentials depicted in Figure S6a shows that MPVN possesses a larger potential than MVN, PVN and NCNF, which can be attributed to reduction of long-chain soluble Li_2_S_x_ (6 < x < 8) to short-chain Li_2_S_x_ (4 < x < 6). Besides, Tafel plots, which are an effective tactic for identifying the catalytic effect of VN on the conversion of lithium polysulfides, showed that MPVN had a decreased Tafel slope of 52 mV dec^−1^, compared to 55, 58 and 65 mV dec^−1^ of MVN, PVN and NCNF, respectively, further confirming the accelerated redox kinetics of lithium polysulfides (Figure S6b). EIS was also measured before and after cycle tests to further analyze the redox kinetics. The Nyquist plots (Figure S7a) of Li–S batteries before cycling consisted of a semicircle (high frequency region) and a straight line (low frequency part). The semicircle (high frequency region) corresponds to charge transfer resistance (R_ct_) within cathodes [64]. The R_ct_ of MPVN (30 Ω cm^−2^) was evidently lower than those of MVN (39 Ω cm^−2^), PVN (56 Ω cm^−2^) and NCNF (77 Ω cm^−2^), indicating the enhanced redox kinetics of MPVN. The slope of the short line (low frequency region) is related to the solid-state diffusion process of Li^+^ into the electrode materials, where the smaller slope of MPVN indicates increased ion diffusion efficiency. After 3 cycles, the Nyquist plots had two semicircles in the high and medium frequency regions and a short line in the low frequency region (Figure S7b). The semicircle in the high frequency region corresponds to Li^+^ diffusion through the surface of nanostructure, which appears after cycling because of the formation of insulated Li_2_S_2_/Li_2_S on the surface or near-surface of the electrode materials. The semicircle in medium frequency may be ascribed to charge transfer and double layer capacitance. The R_ct_ decreases to 5 Ω cm^−2^ for MPVN after 3 CV cycles, which may be ascribed to activation of homogenous sulfur diffusion on the current collector, penetration of electrolyte into the electrode and increased electrochemical contact area.

The GCD (0.1 C rate) of LSBs with potential from 1.8 to 2.8 V was also tested, in which the typical multi-plateau behavior was consistent with the CV tests. The initial discharge capacity with MPVN was 1392 mAh g^−1^, which was much higher than those of MVN (1294 mAh g^−1^), PVN (1021 mAh g^−1^) and NCNF (516 mAh g^−1^), indicating a highly reversible solid–liquid–solid reaction of S↔Li_2_S_x_ (4 ≤ x ≤ 8)↔Li_2_S_2_/Li_2_S. Notably, LSBs with MPVN had the lowest polarization (0.25 V), indicative of fast sulfur-based active material electrochemical dynamics. The GCD measurement can be also applied to analyze the self-discharge rate resulting from the soluble Li_2_S_x_ shuttle effect, which can damage the lifespan of Li–S batteries. Accordingly, soluble Li_2_S_x_ content reached a maximum when the discharging potential was set to 2.1V. After 19 stable cycles, the discharge process was interrupted at about 2.1 V for 48 h, where Li_2_S_x_ diffused and then reduced to Li_2_S_2_/Li_2_S, leading to capacity decay (Figures S8a, S9a and S10a). LSBs with MPVN (Figure S8b) had almost no capacity loss, which was obviously superior than those of MVN (10.28 of △D as decreased discharge capacity, 17.16 of △C as decreased charge capacity in Figure S9b) and PVN (21.09 of △D, 29.43 of △C in Figure S10b). The low capacity loss of MPVN suggests a strong anchoring effect on lithium polysulfides, so as to mitigate the shuttle effect. 

Symmetric cells were also assembled by sandwiching the current collector containing Li_2_S_6_ without lithium foil to further investigate the catalytic kinetics of VN. CV curves of MPVN in Figure 4c exhibit a cathodic peak at –0.36 V, where the Li_2_S_6_ is firstly reduced to Li_2_S_2_/Li_2_S on the working electrode for the first cycle; and an anodic peak at 0.37 V, related to the multistep oxidation of Li_2_S_2_/Li_2_S to higher order Li_2_S_x_ and then conversion to S_8_ on the counter electrode. The high overlap of the subsequent cycle indicates a highly reversible electrochemical conversion of the lithium polysulfides (Figure S11). Moreover, higher current density of Li–S batteries with MPVN is observed compared to other S host materials, indicating an accelerated dynamic redox reaction ascribable to the excellent catalytic effect of MPVN. This was further verified by EIS, shown in Figure 4d, where a semicircle in the high frequency region belonged to charge transfer resistance (R_ct_). The symmetric cells with MPVN exhibited a small R_ct_ of 16 Ω cm^−2^, compared to the 20 Ω cm^−2^ of MVN, 48 Ω cm^−2^ of PVN and 83 Ω cm^−2^ of NCNF, suggesting an enhanced redox dynamic by rapid charge transportation in the electrochemical process. MPVN not only provides strong affinity capability with lithium polysulfides to promote S utilization, it also catalyzes the conversion of lithium polysulfides, enabling enhanced redox kinetics.

**Figure 5:**
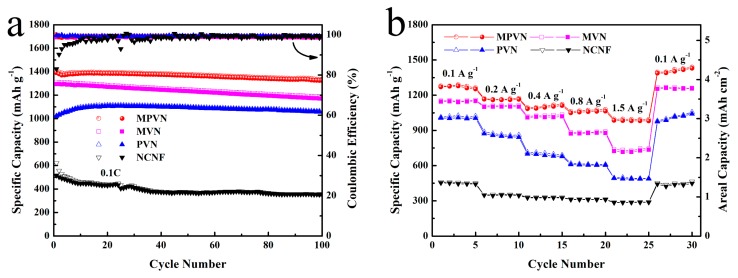
(**a**) Long-term cycling curves at 0.1 C and (**b**) rate capability at various current densities (from 0.1 to 1.5 A g^−1^).

Long-term cycling stability was also an essential factor to evaluate the catalytic stability of VN, and was conducted in coin cells with MPVN, MVN, PVN and NCNF current collectors (Figure 5a). For MPVN-based LSBs, MVPN delivered an initial discharge specific capacity of 1392 mAh g^−1^ (458 Wh kg^−1^ based on MPVN and Li_2_S_6_), and 1325 mAh g^−1^ (436 Wh kg^−1^) was maintained after 100 cycles (high capacity retention of 95.2% and low capacity decay of 0.05% per cycle), superior to those of MVN (0.09%), PVN (0.06%) and NCNF (0.31%). This is because there are more exposed catalytic sites on MPVN to enhance the contact area between VN and lithium polysulfides. Moreover, the coulombic efficiency of MPVN was more stable (about 99%) compared to the other host materials, further confirming its excellent catalytic ability to restrict the shuttle effect of lithium polysulfides. It should be noted that the bare MPVN without Li_2_S_6_ active materials displayed a low specific capacity of merely 39 mAh g^−1^ for lithium storage (Figure S13), confirming the capacity in the lithium–sulfur system is mainly attributable to the redox reaction of S species.

As shown in Figure 5b, the reversible discharge capacities of LSBs with MPVN were 1288, 1154, 1111, 1064 and 985 mAh g^−1^ at various current densities (0.1 to 1.5 A g^−1^). In contrast, the batteries with MVN and PVN displayed a poorer rate performance than MPVN, related to their fewer catalytic sites for VN decorating. Without VN, NCNF showed the lowest electrochemical performance, further confirming the vital vole of VN in the lithium–sulfur system. Demonstrating the synergistic effect between the appropriate porous channel design and the VN catalytic effect, LSBs with MPVN exhibited an obvious S electrochemical reaction plateau, even at a high current density of 1.5 A g^−1^ (Figure S12). As depicted in Figure S13a, batteries with MPVN still delivered a higher specific capacity—1017 mAh g^−1^ (6.1 mAh cm^−2^)—even at a high sulfur loading of 6 mg cm^−2^, which is higher than the 4 mAh cm^−2^ of commercial Li-ion batteries. After 200 cycles, the LSBs with MPVN still delivered a high capacity, retaining 811 mAh g^−1^, and high capacity retention (80%) (Figure S14b). The attractive energy storage of LSBs enhanced by MPVN further confirms the strong affinity and catalytic effect with lithium polysulfides, realizing the fast redox kinetics of S species for superior Li–S energy systems.

## 4. Conclusions 

Herein, we designed a lotus root-like NCNF structure assembled with VN catalysts as a self-supported current collector for superior LSBs. In the experimental host material, MPVN not only trapped lithium polysulfides by strong chemical affinity but also electro-catalyzed the conversion of S↔Li_2_S_x_↔Li_2_S_2_/Li_2_S, thereby realizing fast redox kinetics and promoting the utilization of S for high cycling capability and rate performance. Furthermore, the multichannel porous nanostructure was beneficial for Li ion transfer and as an electrolyte–electrode contact interface. LSBs with an MPVN current collector displayed a high reversible capacity of 1325 mAh g^−1^ at 0.1 C, with a high capacity retention of 95% and excellent rate performance (985 mAh g^−1^ at 1.5 A g^−1^).

## Supplementary Materials

The following are available online at https://www.mdpi.com/2079-4991/9/12/1724/s1, Figure S1: Photograph of MPVN after annealed at 800 °C. Figure S2: Morphology of MVN. (a,b) SEM. (c,d) TEM. Figure S3: Morphology of PVN. (a,b) SEM. (c,d) TEM. Figure S4: XPS spectra of (a) V 2p, (b) N 1s, (c) C1s and (d) S2p in MVN before and after Li_2_S_6_ adsorption. Figure S5: XPS spectra of (a) V 2p, (b) N 1s, (c) C1s and (d) S2p in PVN before and after Li_2_S_6_ adsorption. Figure S6: (a) Potentiostatic polarization curves of MPVN, MVN and PVN at a scan rate of 0.05 mV s^−1^. (b) Tafel plots derived of potentiostatic polarization curves. Figure S7: Nyquist plots of MPVN, MVN and PVN (a) before and (b) after CV cycles. Figure S8: Self-discharge experiments: voltage profiles of the 19th cycle (complete discharge) and 20th cycle (after 24 h at discharge) for MPVN. Figure S9: Self-discharge curves of MVN. Figure S10: Self-discharge curves of PVN. Figure S11: curves of symmetric cells with MPVN. Figure S12: GCD curves of MPVN at different current density. Figure S13: Capacity contribution of MPVN without active materials sulfur at 0.1 C. Figure S14: (a) GCD curves at second cycle and (b) long-term cycle curves of MPVN with a high sulfur loading of 6 mg cm^−2^ at 0.1 C.

## References

[1] Seh Z.W., Sun Y., Zhang Q., Cui Y. (2016). Designing high-energy lithium-sulfur batteries. Chem. Soc. Rev..

[2] Lochala J., Liu D., Wu B., Robinson C., Xiao J. (2017). Research Progress toward the Practical Applications of Lithium-Sulfur Batteries. ACS Appl. Mater. Interfaces.

[3] Xu J., Ma J., Fan Q., Guo S., Dou S. (2017). Recent Progress in the Design of Advanced Cathode Materials and Battery Models for High-Performance Lithium-X (X = O_2_, S, Se, Te, I_2_, Br_2_) Batteries. Adv. Mater..

[4] Lv D., Zheng J., Li Q., Xie X., Ferrara S., Nie Z., Mehdi L.B., Browning N.D., Zhang J.-G., Graff G.L. (2015). High Energy Density Lithium-Sulfur Batteries: Challenges of Thick Sulfur Cathodes. Adv. Energy Mater..

[5] Fang R., Zhao S., Sun Z., Wang D.W., Cheng H.M., Li F. (2017). More Reliable Lithium-Sulfur Batteries: Status, Solutions and Prospects. Adv. Mater..

[6] Kuzmina E.V., Karaseva E.V., Kolosnitsyn D.V., Sheina L.V., Shakirova N.V., Kolosnitsyn V.S. (2018). Sulfur redistribution between positive and negative electrodes of lithium-sulfur cells during cycling. J. Power Sources.

[7] Li Y., Romero N.A., Lau K.C. (2018). Structure-Property of Lithium-Sulfur Nanoparticles via Molecular Dynamics Simulation. ACS Appl. Mater. Interfaces.

[8] Shen X., Qian T., Chen P., Liu J., Wang M., Yan C. (2018). Bioinspired Polysulfiphobic Artificial Interphase Layer on Lithium Metal Anodes for Lithium Sulfur Batteries. ACS Appl. Mater. Interfaces.

[9] Qu H.T., Ju J.W., Chen B.B., Xue N., Du H.P., Han X.Q., Zhang J.J., Xu G.J., Yu Z., Wang X.G. (2018). Inorganic separators enable significantly suppressed polysulfide shuttling in high-performance lithium-sulfur batteries. J. Mater. Chem. A.

[10] Zang Y., Pei F., Huang J.H., Fu Z.H., Xu G., Fang X.L. (2018). Large-Area Preparation of Crack-Free Crystalline Microporous Conductive Membrane to Upgrade High Energy Lithium-Sulfur Batteries. Adv. Energy Mater..

[11] Tian W., Xi B., Mao H., Zhang J., Feng J., Xiong S. (2018). Systematic Exploration of the Role of a Modified Layer on the Separator in the Electrochemistry of Lithium-Sulfur Batteries. ACS Appl. Mater. Interfaces.

[12] Guo Y., Sun M., Liang H., Ying W., Zeng X., Ying Y., Zhou S., Liang C., Lin Z., Peng X. (2018). Blocking Polysulfides and Facilitating Lithium-Ion Transport: Polystyrene Sulfonate@ HKUST-1 Membrane for Lithium-Sulfur Batteries. ACS Appl. Mater. Interfaces.

[13] Song X., Chen G., Wang S., Huang Y., Jiang Z., Ding L.X., Wang H. (2018). Self-Assembled Close-Packed MnO_2_ Nanoparticles Anchored on a Polyethylene Separator for Lithium-Sulfur Batteries. ACS Appl. Mater. Interfaces.

[14] Song J., Su D., Xie X., Guo X., Bao W., Shao G., Wang G. (2016). Immobilizing Polysulfides with MXene-Functionalized Separators for Stable Lithium-Sulfur Batteries. ACS Appl. Mater. Interfaces.

[15] Zhang G., Peng H.J., Zhao C.Z., Chen X., Zhao L.D., Li P., Huang J.Q., Zhang Q. (2018). The Radical Pathway Based on a Lithium-Metal-Compatible High-Dielectric Electrolyte for Lithium-Sulfur Batteries. Angew. Chem. Int. Edit..

[16] Lang S.Y., Xiao R.J., Gu L., Guo Y.G., Wen R., Wan L.J. (2018). Interfacial Mechanism in Lithium-Sulfur Batteries: How Salts Mediate the Structure Evolution and Dynamics. J. Am. Chem. Soc..

[17] Zheng D., Wang G., Liu D., Harris J.B., Ding T., Si J., Qu D., Yang X.-Q., Qu D. (2018). Systematic and rapid screening for the redox shuttle inhibitors in lithium-sulfur batteries. Electrochim. Acta.

[18] Chen Z., Zhou J., Guo Y., Liang C., Yang J., Wang J., Nuli Y. (2018). A compatible carbonate electrolyte with lithium anode for high performance lithium sulfur battery. Electrochim. Acta.

[19] Yang W., Yang W., Sun B., Di S., Yan K., Wang G., Shao G. (2018). Mixed Lithium Oxynitride/Oxysulfide as an Interphase Protective Layer to Stabilize Lithium Anodes for High-Performance Lithium-Sulfur Batteries. ACS Appl. Mater. Interfaces.

[20] Cao R., Xu W., Lv D., Xiao J., Zhang J.-G. (2015). Anodes for Rechargeable Lithium-Sulfur Batteries. Adv. Energy Mater..

[21] Cao R., Chen J., Han K.S., Xu W., Mei D., Bhattacharya P., Engelhard M.H., Mueller K.T., Liu J., Zhang J.-G. (2016). Effect of the Anion Activity on the Stability of Li Metal Anodes in Lithium-Sulfur Batteries. Adv. Funct. Mater..

[22] Pang Q., Kwok C.Y., Kundu D., Liang X., Nazar L.F. (2019). Lightweight Metallic MgB_2_ Mediates Polysulfide Redox and Promises High-Energy-Density Lithium-Sulfur Batteries. Joule.

[23] Chen X., Yuan L., Hao Z., Liu X., Xiang J., Zhang Z., Huang Y., Xie J. (2018). Free-Standing Mn_3_O_4_@CNF/S Paper Cathodes with High Sulfur Loading for Lithium-Sulfur Batteries. ACS Appl. Mater. Interfaces.

[24] Zhou T.H., Lv W., Li J., Zhou G.M., Zhao Y., Fan S.X., Liu B.L., Li B.H., Kang F.Y., Yang Q.H. (2017). Twinborn TiO_2_-TiN heterostructures enabling smooth trapping-diffusion-conversion of polysulfides towards ultralong life lithium-sulfur batteries. Energy Environ. Sci..

[25] Yu M., Wang Z., Wang Y., Dong Y., Qiu J. (2017). Freestanding Flexible Li_2_S Paper Electrode with High Mass and Capacity Loading for High-Energy Li-S Batteries. Adv. Energy Mater..

[26] An Y., Zhang Z., Fei H., Xiong S., Ji B., Feng J. (2017). Ultrafine TiO_2_ Confined in Porous-Nitrogen-Doped Carbon from Metal-Organic Frameworks for High-Performance Lithium Sulfur Batteries. ACS Appl. Mater. Interfaces.

[27] Li X., Guo G., Qin N., Deng Z., Lu Z., Shen D., Zhao X., Li Y., Su B.L., Wang H.E. (2018). SnS_2_/TiO_2_ nanohybrids chemically bonded on nitrogen-doped graphene for lithium-sulfur batteries: Synergy of vacancy defects and heterostructures. Nanoscale.

[28] Feng G., Liu X., Liu Y., Wu Z., Chen Y., Guo X., Zhong B., Xiang W., Li J. (2018). Trapping polysulfides by chemical adsorption barrier of Li_x_La_y_TiO_3_ for enhanced performance in lithium-sulfur batteries. Electrochim. Acta.

[29] Li H., Sun L., Zhao Y., Tan T., Zhang Y. (2019). Blackberry-like hollow graphene spheres synthesized by spray drying for high-performance lithium-sulfur batteries. Electrochim. Acta.

[30] Pongilat R., Nallathamby K. (2018). Electrocatalysis of Ruthenium Nanoparticles-Decorated Hollow Carbon Spheres for the Conversion of Li_2_S_2_/Li_2_S in Lithium-Sulfur Batteries. ACS Appl. Mater. Interfaces.

[31] Yin Y., Franco A.A. (2018). Unraveling the Operation Mechanisms of Lithium Sulfur Batteries with Ultramicroporous Carbons. ACS Appl. Energy Mater..

[32] Yu J.J., Li X.L., Shu Y.Z., Ma L., Zhang X.C., Ding Y.S. (2019). Anchoring polysulfides in hierarchical porous carbon aerogel via electric-field-responsive switch for lithium sulfur battery. Electrochim. Acta.

[33] Liu T., Sun S.M., Song W., Sun X.L., Niu Q.H., Liu H., Ohsaka T., Wu J.F. (2018). A lightweight and binder-free electrode enabled by lignin fibers@ carbon-nanotubes and graphene for ultrastable lithium-sulfur batteries. J. Mater. Chem. A.

[34] Guo Z., Nie H., Yang Z., Hua W., Ruan C., Chan D., Ge M., Chen X., Huang S. (2018). 3D CNTs/Graphene-S-Al_3_Ni_2_ Cathodes for High-Sulfur-Loading and Long-Life Lithium-Sulfur Batteries. Adv. Sci..

[35] Deng N., Kang W., Ju J., Fan L., Zhuang X., Ma X., He H., Zhao Y., Cheng B. (2017). Polyvinyl Alcohol-derived carbon nanofibers/carbon nanotubes/sulfur electrode with honeycomb-like hierarchical porous structure for the stable-capacity lithium/sulfur batteries. J. Power Sources.

[36] Kang W., Fan L., Deng N., Zhao H., Li Q., Naebe M., Yan J., Cheng B. (2018). Sulfur-embedded porous carbon nanofiber composites for high stability lithium-sulfur batteries. Chem. Eng. J..

[37] Deng W., Hu A., Chen X., Zhang S., Tang Q., Liu Z., Fan B., Xiao K. (2016). Sulfur-impregnated 3D hierarchical porous nitrogen-doped aligned carbon nanotubes as high-performance cathode for lithium-sulfur batteries. J. Power Sources.

[38] Pan H., Chen J., Cao R., Murugesan V., Rajput N.N., Han K.S., Persson K., Estevez L., Engelhard M.H., Zhang J.-G. (2017). Non-encapsulation approach for high-performance Li–S batteries through controlled nucleation and growth. Nat. Energy.

[39] Liang Y., Deng N., Ju J., Zhou X., Yan J., Zhong C., Kang W., Cheng B. (2018). Facilitation of lithium polysulfides adsorption by nitrogen doped carbon nanofibers with 3D interconnected pore structures for high-stable lithium-sulfur batteries. Electrochim. Acta.

[40] Wu H., Xia L., Ren J., Zheng Q., Xie F., Jie W., Xu C., Lin D. (2018). A multidimensional and nitrogen-doped graphene/hierarchical porous carbon as a sulfur scaffold for high performance lithium sulfur batteries. Electrochim. Acta.

[41] Wu K.S., Hu Y., Shen Z., Chen R.Z., He X., Cheng Z.L., Pan P. (2018). Highly efficient and green fabrication of a modified C nanofiber interlayer for high-performance Li-S batteries. J. Mater. Chem. A.

[42] Liu X., Huang J.Q., Zhang Q., Mai L. (2017). Nanostructured Metal Oxides and Sulfides for Lithium-Sulfur Batteries. Adv. Mater..

[43] He J.R., Hartmann G., Lee M., Hwang G.S., Chen Y.F., Manthiram A. (2019). Freestanding 1T MoS_2_/graphene heterostructures as a highly efficient electrocatalyst for lithium polysulfides in Li-S batteries. Energy Environ. Sci..

[44] Cai D., Wang L., Li L., Zhang Y., Li J., Chen D., Tu H., Han W. (2019). Self-assembled CdS quantum dots in carbon nanotubes: Induced polysulfide trapping and redox kinetics enhancement for improved lithium–sulfur battery performance. J. Mater. Chem. A.

[45] Li S., Cen Y., Xiang Q., Aslam M.K., Hu B., Li W., Tang Y., Yu Q., Liu Y., Chen C. (2019). Vanadium dioxide–reduced graphene oxide binary host as an efficient polysulfide plague for high-performance lithium–sulfur batteries. J. Mater. Chem. A.

[46] Wang S., Liao J., Yang X., Liang J., Sun Q., Liang J., Zhao F., Koo A., Kong F., Yao Y. (2019). Designing a highly efficient polysulfide conversion catalyst with paramontroseite for high-performance and long-life lithium-sulfur batteries. Nano Energy.

[47] Ali S., Waqas M., Jing X., Chen N., Chen D., Xiong J., He W. (2018). Carbon-Tungsten Disulfide Composite Bilayer Separator for High-Performance Lithium-Sulfur Batteries. ACS Appl. Mater. Interfaces.

[48] Ma L., Zhang W., Wang L., Hu Y., Zhu G., Wang Y., Chen R., Chen T., Tie Z., Liu J. (2018). Strong Capillarity, Chemisorption, and Electrocatalytic Capability of Crisscrossed Nanostraws Enabled Flexible, High-Rate, and Long-Cycling Lithium-Sulfur Batteries. ACS Nano.

[49] Song Y., Wang H., Yu W.S., Wang J.X., Liu G.X., Li D., Wang T.T., Yang Y., Dong X.T., Ma Q.L. (2018). Synergistic stabilizing lithium sulfur battery via nanocoating polypyrrole on cobalt sulfide nanobox. J. Power Sources.

[50] Li W., Hicks-Garner J., Wang J., Liu J., Gross A.F., Sherman E., Graetz J., Vajo J.J., Liu P. (2014). V_2_O_5_ Polysulfide Anion Barrier for Long-Lived Li-S Batteries. Chem. Mater..

[51] Yuan Z., Peng H.J., Hou T.Z., Huang J.Q., Chen C.M., Wang D.W., Cheng X.B., Wei F., Zhang Q. (2016). Powering Lithium-Sulfur Battery Performance by Propelling Polysulfide Redox at Sulfiphilic Hosts. Nano Lett..

[52] Wang H.E., Yin K., Zhao X., Qin N., Li Y., Deng Z., Zheng L., Su B.L., Lu Z. (2018). Coherent TiO_2_/BaTiO_3_ heterostructure as a functional reservoir and promoter for polysulfide intermediates. Chem. Commun..

[53] Ni J., Jin L., Xue M., Zheng J., Zheng J.P., Zhang C. (2019). TiO_2_ microboxes as effective polysufide reservoirs for lithium sulfur batteries. Electrochim. Acta.

[54] He B., Li W.C., Zhang Y., Yu X.F., Zhang B.S., Li F., Lu A.H. (2018). Paragenesis BN/CNTs hybrid as a monoclinic sulfur host for high rate and ultra-long life lithium-sulfur battery. J. Mater. Chem. A.

[55] Ye C., Jiao Y., Jin H., Slattery A.D., Davey K., Wang H., Qiao S.Z. (2018). 2D MoN-VN Heterostructure to Regulate Polysulfides for Highly Efficient Lithium-Sulfur Batteries. Angew. Chem. Int. Edit..

[56] Zhang L., Chen X., Wan F., Niu Z., Wang Y., Zhang Q., Chen J. (2018). Enhanced Electrochemical Kinetics and Polysulfide Traps of Indium Nitride for Highly Stable Lithium-Sulfur Batteries. ACS Nano.

[57] Li Z., He Q., Xu X., Zhao Y., Liu X., Zhou C., Ai D., Xia L., Mai L. (2018). A 3D Nitrogen-Doped Graphene/TiN Nanowires Composite as a Strong Polysulfide Anchor for Lithium-Sulfur Batteries with Enhanced Rate Performance and High Areal Capacity. Adv. Mater..

[58] Liao Y., Xiang J., Yuan L., Hao Z., Gu J., Chen X., Yuan K., Kalambate P.K., Huang Y. (2018). Biomimetic Root-like TiN/C@S Nanofiber as a Freestanding Cathode with High Sulfur Loading for Lithium–Sulfur Batteries. ACS Appl. Mater. Interfaces.

[59] Xing Z.Y., Li G.R., Sy S., Chen Z.W. (2018). Recessed deposition of TiN into N-doped carbon as a cathode host for superior Li-S batteries performance. Nano Energy.

[60] Wang Y., Zhang R., Pang Y.-C., Chen X., Lang J., Xu J., Xiao C., Li H., Xi K., Ding S. (2019). Carbon@titanium nitride dual shell nanospheres as multi-functional hosts for lithium sulfur batteries. Energy Storage Mater..

[61] Jeong T.-G., Choi D.S., Song H., Choi J., Park S.-A., Oh S.H., Kim H., Jung Y., Kim Y.-T. (2017). Heterogeneous Catalysis for Lithium–Sulfur Batteries: Enhanced Rate Performance by Promoting Polysulfide Fragmentations. ACS Energy Lett..

[62] Qi B., Zhao X., Wang S., Chen K., Wei Y., Chen G., Gao Y., Zhang D., Sun Z., Li F. (2018). Mesoporous TiN microspheres as an efficient polysulfide barrier for lithium–sulfur batteries. J. Mater. Chem. A.

[63] Ren W., Xu L., Zhu L., Wang X., Ma X., Wang D. (2018). Cobalt-Doped Vanadium Nitride Yolk-Shell Nanospheres @ Carbon with Physical and Chemical Synergistic Effects for Advanced Li-S Batteries. ACS Appl. Mater. Interfaces.

[64] Song Y.Z., Zhao W., Kong L., Zhang L., Zhu X.Y., Shao Y.L., Ding F., Zhang Q., Sun J.Y., Liu Z.F. (2018). Synchronous immobilization and conversion of polysulfides on a VO_2_-VN binary host targeting high sulfur load Li-S batteries. Energy Environ. Sci..

[65] Zhong Y., Chao D., Deng S., Zhan J., Fang R., Xia Y., Wang Y., Wang X., Xia X., Tu J. (2018). Confining Sulfur in Integrated Composite Scaffold with Highly Porous Carbon Fibers/Vanadium Nitride Arrays for High-Performance Lithium-Sulfur Batteries. Adv. Funct. Mater..

[66] Sun Z., Zhang J., Yin L., Hu G., Fang R., Cheng H.M., Li F. (2017). Conductive porous vanadium nitride/graphene composite as chemical anchor of polysulfides for lithium-sulfur batteries. Nat. Commun..

[67] Ma L., Yuan H., Zhang W., Zhu G., Wang Y., Hu Y., Zhao P., Chen R., Chen T., Liu J. (2017). Porous-Shell Vanadium Nitride Nanobubbles with Ultrahigh Areal Sulfur Loading for High-Capacity and Long-Life Lithium-Sulfur Batteries. Nano Lett..

[68] Lee J.S., Kim W., Jang J., Manthiram A. (2017). Sulfur-Embedded Activated Multichannel Carbon Nanofiber Composites for Long-Life, High-Rate Lithium-Sulfur Batteries. Adv. Energy Mater..

[69] Wang X., Yang C., Xiong X., Chen G., Huang M., Wang J.-H., Liu Y., Liu M., Huang K. (2019). A robust sulfur host with dual lithium polysulfide immobilization mechanism for long cycle life and high capacity Li-S batteries. Energy Storage Mater..

[70] Guan B., Fan L.S., Wu X., Wang P.X., Qiu Y., Wang M.X., Guo Z.K., Zhang N.Q., Sun K.N. (2018). The facile synthesis and enhanced lithium-sulfur battery performance of an amorphous cobalt boride (Co_2_B) @ graphene composite cathode. J. Mater. Chem. A.

[71] Lim W.G., Kim S., Jo C., Lee J. (2019). A Comprehensive Review of Materials with Catalytic Effects in Li-S Batteries: Enhanced Redox Kinetics. Angew. Chem. Int. Ed..

