# Supplementary material for: Lotus Root-Like Nitrogen-Doped Carbon Nanofiber Structure Assembled with VN Catalysts as a Multifunctional Host for Superior Lithium–Sulfur Batteries
Source: Nanomaterials (Basel). 2019 Dec 3;9(12):1724. doi: 10.3390/nano9121724 (PMC6956178; doi:10.3390/nano9121724)
Supplement: Supplementary file 1 [file nanomaterials-09-01724-s001.pdf]

Lotus Root-Like Nitrogen-Doped Carbon Nanofiber Structure Assembled with VN Catalysts as a Multifunctional Host for Superior Lithium–Sulfur Batteries

Benben Wei [1,2], Chaoqun Shang [1,2,*], Xiaoying Pan [2], Zhihong Chen [3], Lingling Shui [2], Xin Wang [1,2, *] and Guofu Zhou [1,2]

[1] International Academy of Optoelectronics at Zhaoqing, South China Normal University, Zhaoqing 526238, China

[2] National Center for International Research on Green Optoelectronics, South China Normal University, Guangzhou 510006, China

[3] Key Laboratory for Water Quality and Conservation of the Pearl River Delta, Guangzhou University, Guangzhou 510006, China.

* Correspondence: chaoqun.shang@ecs-scnu.org (C.S.); wangxin@scnu.edu.cn (X.W.)

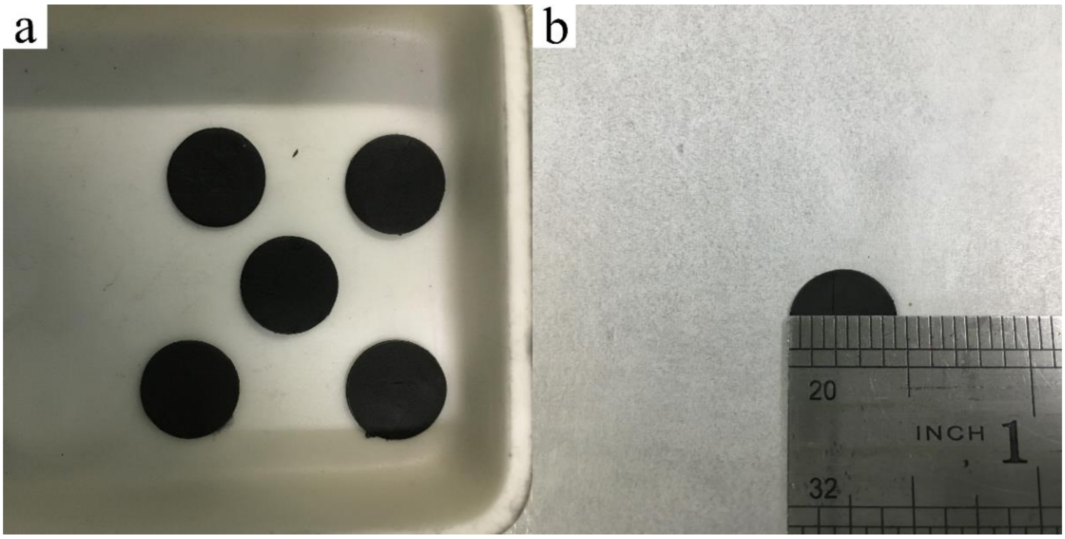

Figure S1. Photograph of MPVN after annealed at 800 °C.

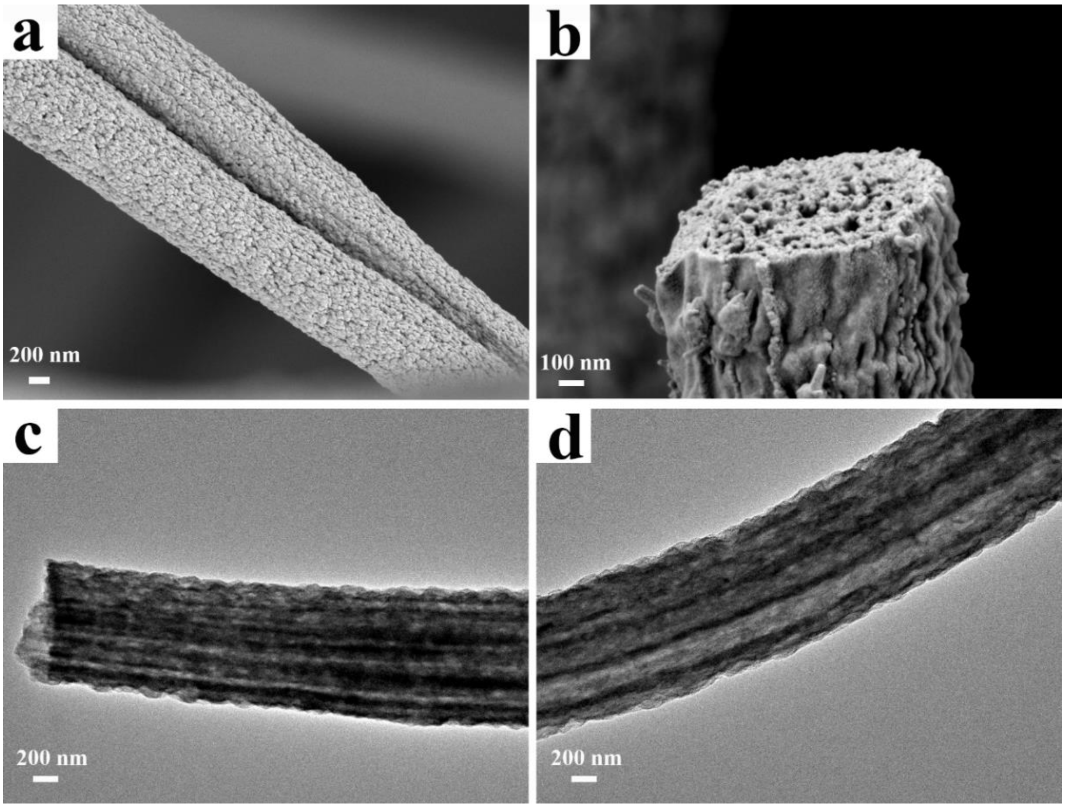

Figure S2. Morphology of MVN. (**a**)–(**b**) SEM. (**c**)–(**d**) TEM.

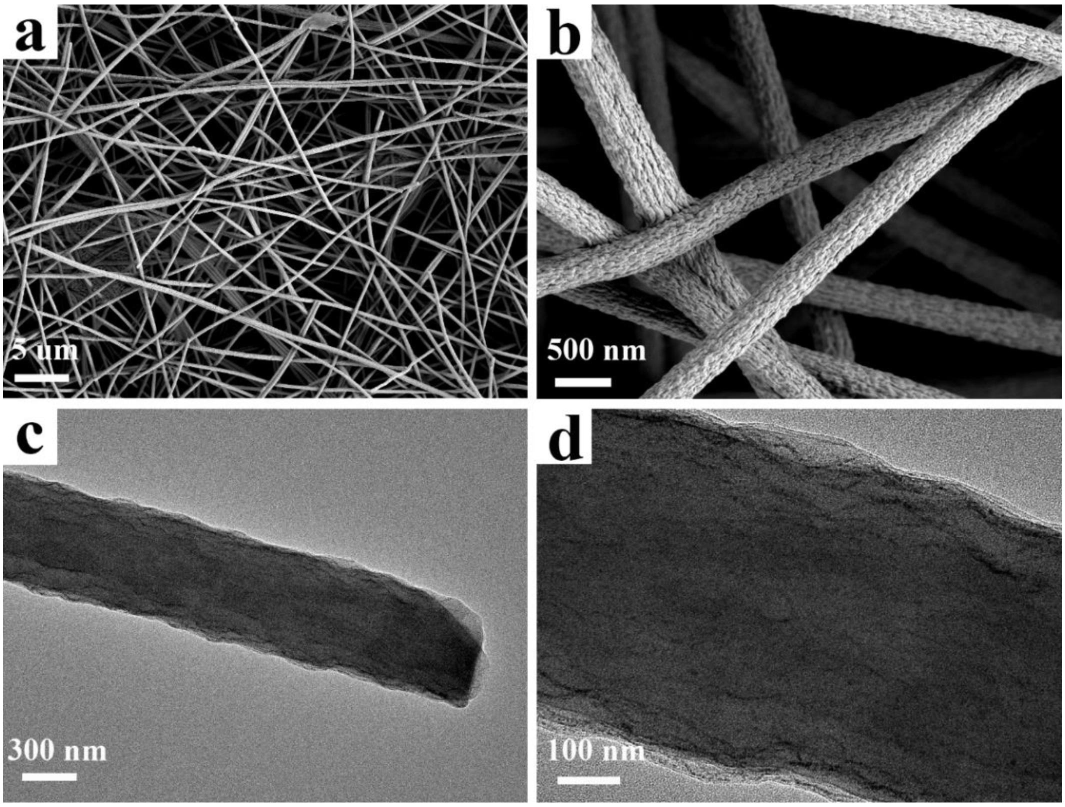

Figure S3. Morphology of PVN. (**a**)–(**b**) SEM. (**c**)–(**d**) TEM.

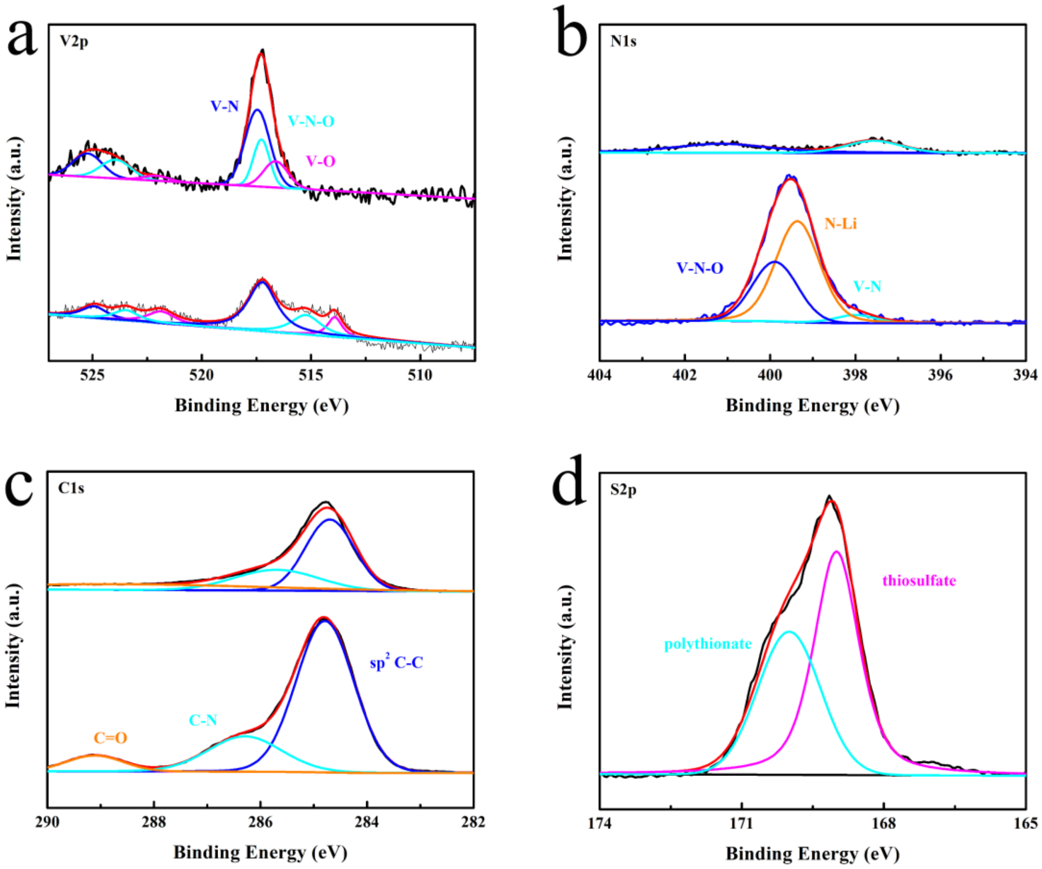

Figure S4. XPS spectra of (**a**) V 2p, (**b**) N 1s, (**c**) C1s and (**d**) S2p in MVN before and after Li_2S_6 adsorption.

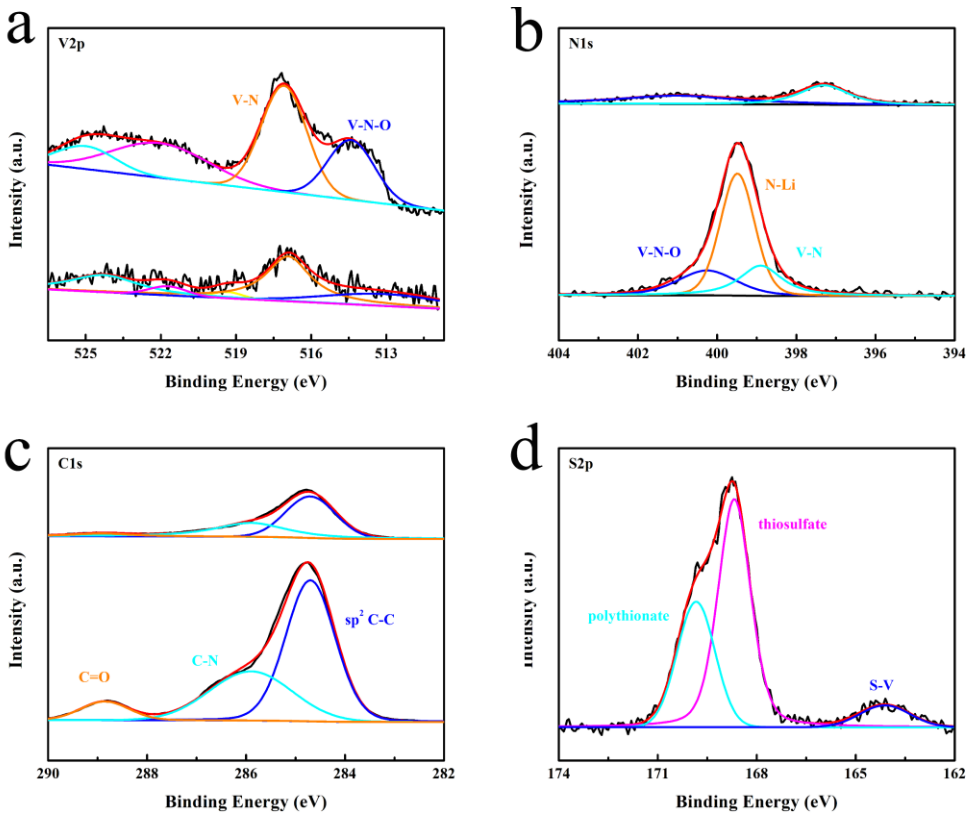

Figure S5. XPS spectra of (**a**) V 2p, (**b**) N 1s, (**c**) C1s and (**d**) S2p in PVN before and after Li_2S_6 adsorption.

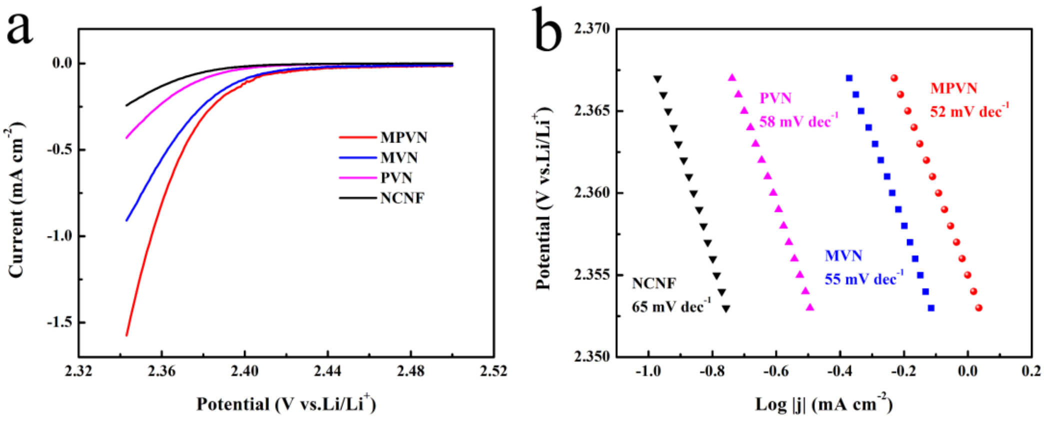

Figure S6. (**a**) Potentiostatic polarization curves of MPVN, MVN and PVN at a scan rate of 0.05 mV s^{-1}. (**b**) Tafel plots derived of potentiostatic polarization curves.

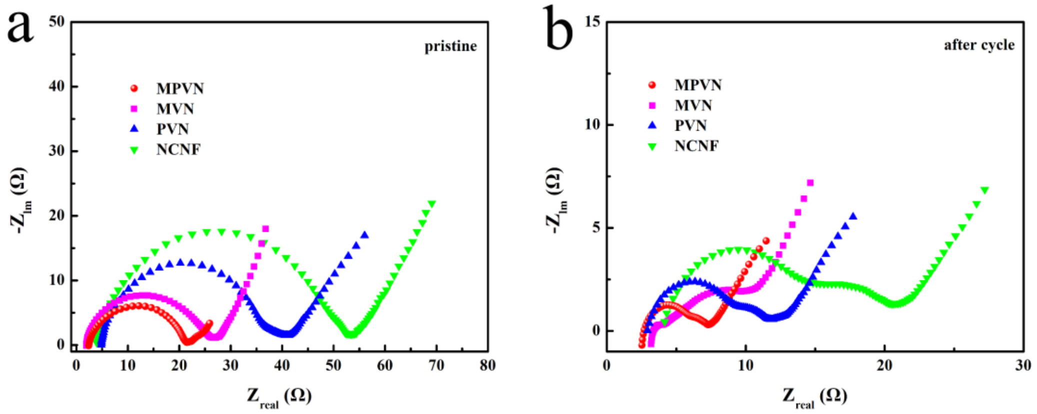

Figure S7. Nyquist plots of MPVN, MVN and PVN (**a**) before and (**b**) after CV cycles.

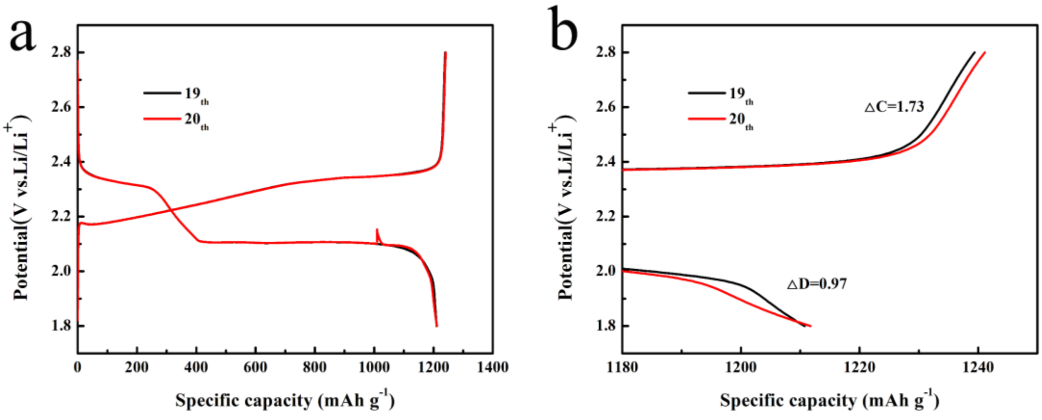

Figure S8. Self-discharge experiments: voltage profiles of the 19th cycle (complete discharge) and 20th cycle (after 24h at discharge) for MPVN.

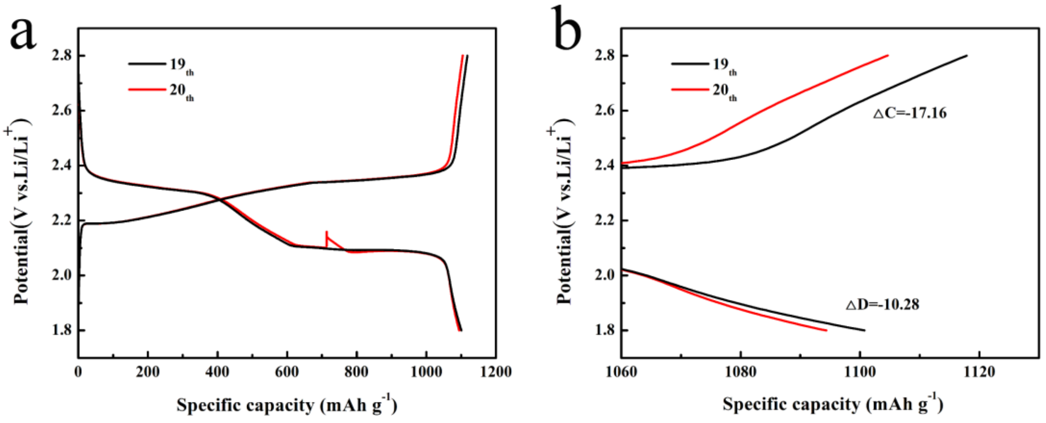

Figure S9. Self-discharge curves of MVN.

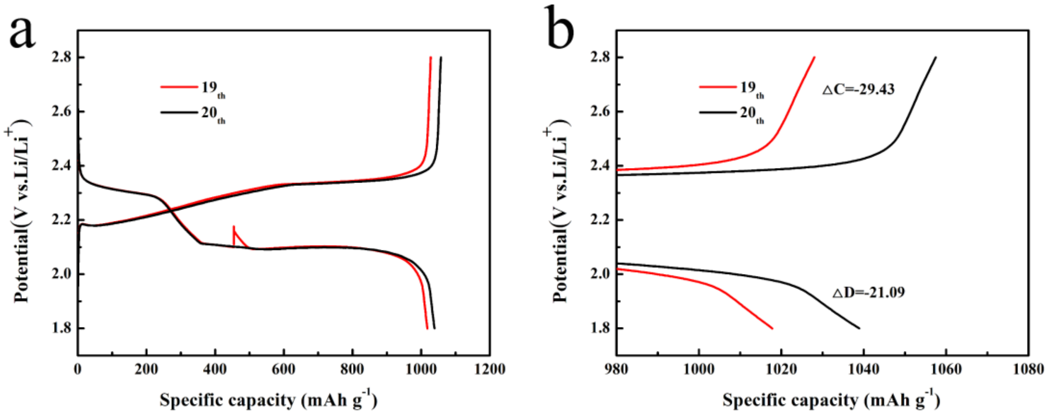

Figure S10. Self-discharge curves of PVN.

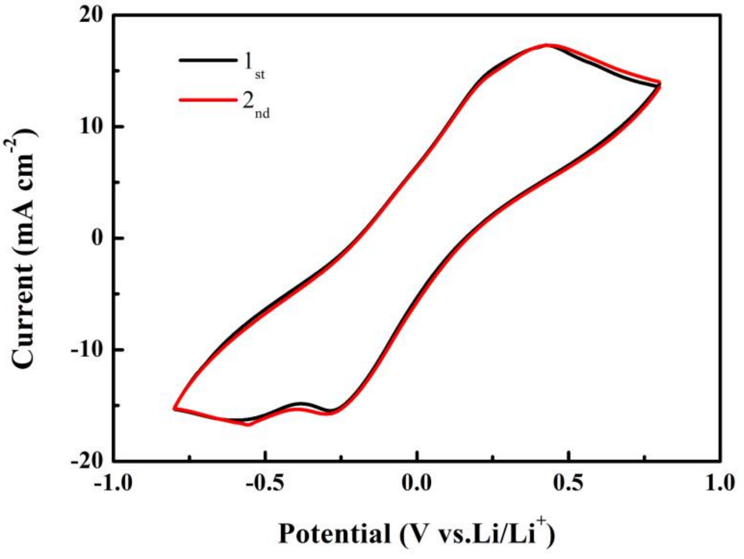

Figure S11. curves of symmetric cells with MPVN.

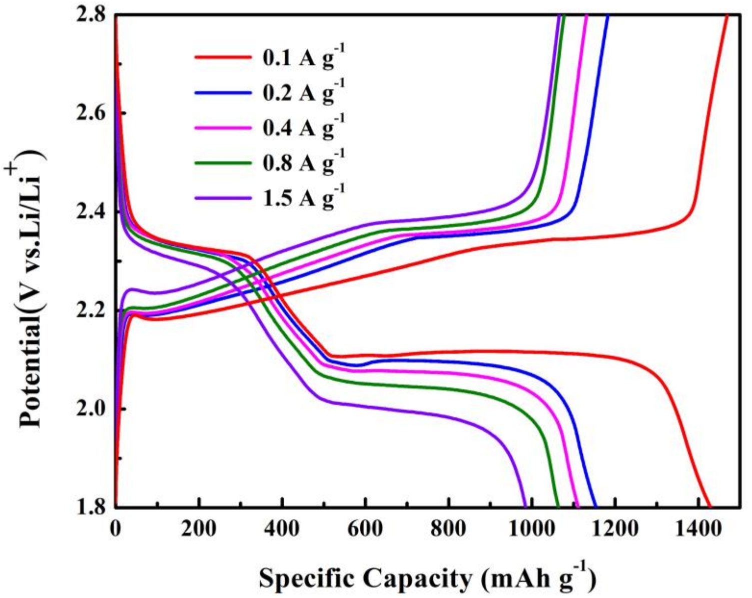

Figure S12. GCD curves of MPVN at different current density.

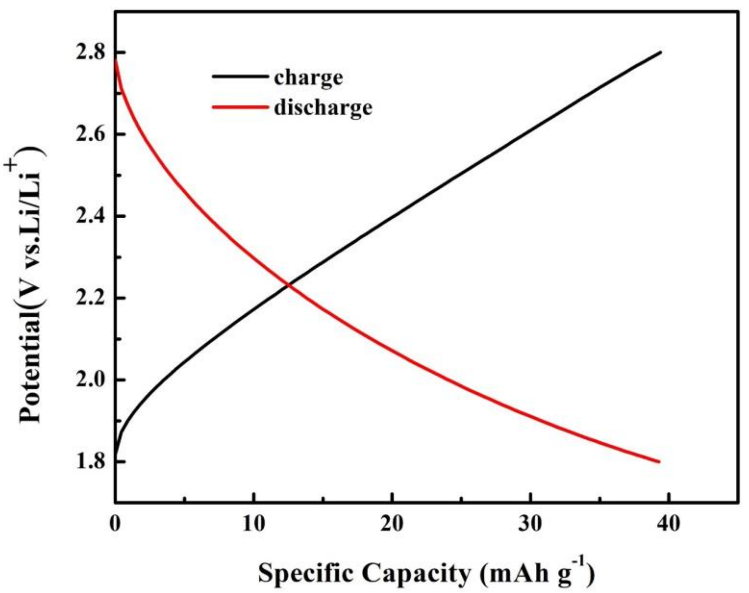

Figure S13. Capacity contribution of MPVN without active materials sulfur at 0.1 C.

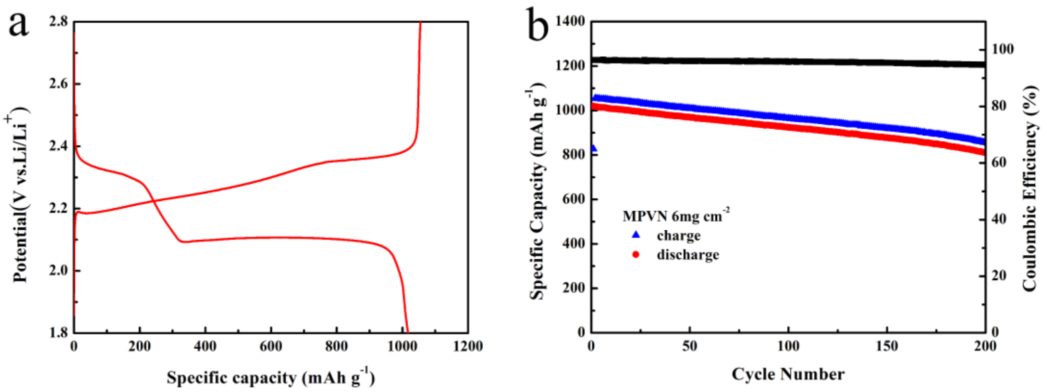

Figure S14. (**a**) GCD curves at second cycle and (**b**) long-term cycle curves of MPVN with a high sulfur loading of 6 mg cm^{-2} at 0.1 C.
